# Removal of large hydatid cysts with balloon-assisted modification of Dowling’s method: technical report

**DOI:** 10.1007/s00701-015-2449-x

**Published:** 2015-05-17

**Authors:** Murat Ulutas, Kadir Cinar, Mehmet Secer

**Affiliations:** Department of Neurosurgery, Konukoglu Hospital, Sanko University, Gaziantep, Turkey; Department of Neurosurgery, Deva Hospital, Gaziantep, Turkey; Department of Neurosurgery, Ali Fuat cebesoy Bulvari Sanko University Konukoglu Hospital, Şehitkamil 27090 Gaziantep, Turkey

**Keywords:** Surgical technic, Cyst hydatid, Intracerebral, New technique, Balloon assisted, Dowling’s method

## Abstract

**Background:**

Delivery of hydatid cysts, especially large ones, without rupture is very important and there is still no 100 % successful method.

**Methods:**

After the hydatid cyst was reached, starting near the surface working around the cyst toward the base, a Foley probe was advanced and, in the region of desired dissection, the balloon of the Foley probe was inflated, and adhesion bands were freed to allow dissection.

**Conclusions:**

We believe our balloon-aided dissection technique is a method that increases the chances of delivering hydatid cysts, with no calcification and secondary infection, without rupture.

**Electronic supplementary material:**

The online version of this article (doi:10.1007/s00701-015-2449-x) contains supplementary material, which is available to authorised users.

## Introduction

It is very important to remove cerebral hydatid cysts without rupture, especially those of large size. Wide scalp flap and dissection of the atrophic cortex, lowering the head of the operating table to benefit from gravity and the use of warm saline between the hydatid cyst and brain parenchyma to deliver the hydatid cyst is the most frequently used method, known as Dowling’s technique [1], with an 88 % success rate reported [2]. The rupture of the cyst may allow spread of the parasite and cause an anaphylactic shock tableau that may result in death and, in spite of medical treatment, repeated surgical interventions may be required for newly-formed cysts [3].

**Fig. 1:**
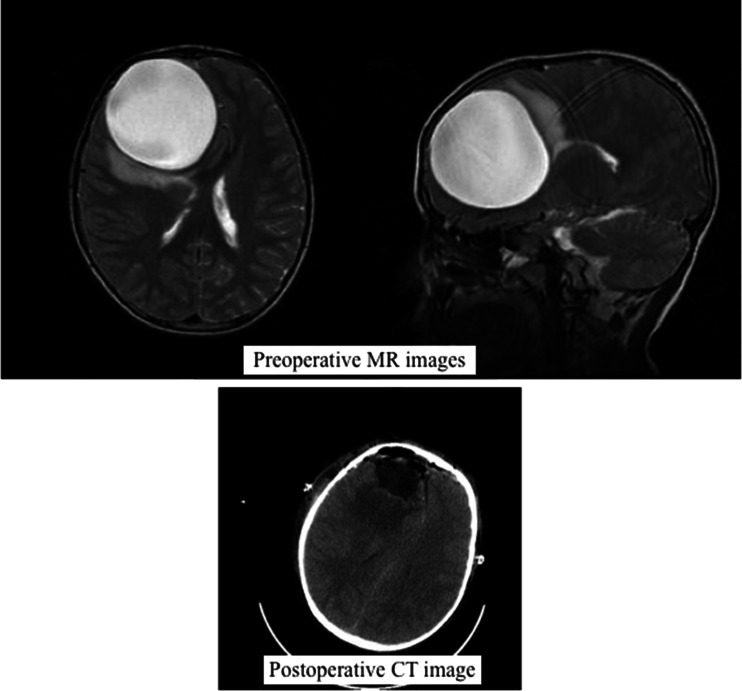
T2-weighted magnetic resonance (MR) image of a cystic lesion in accordance with a hydatid cyst, 7 cm in diameter, located in the right frontal lobe, exceeding the frontal cortex, with smooth edges, filled with homogeneous fluid and with shift and compression effects. Cranial CT check after removal of frontal hydatid cyst, showing reduction in cyst location and in compression effects of the cyst

**Fig. 2:**
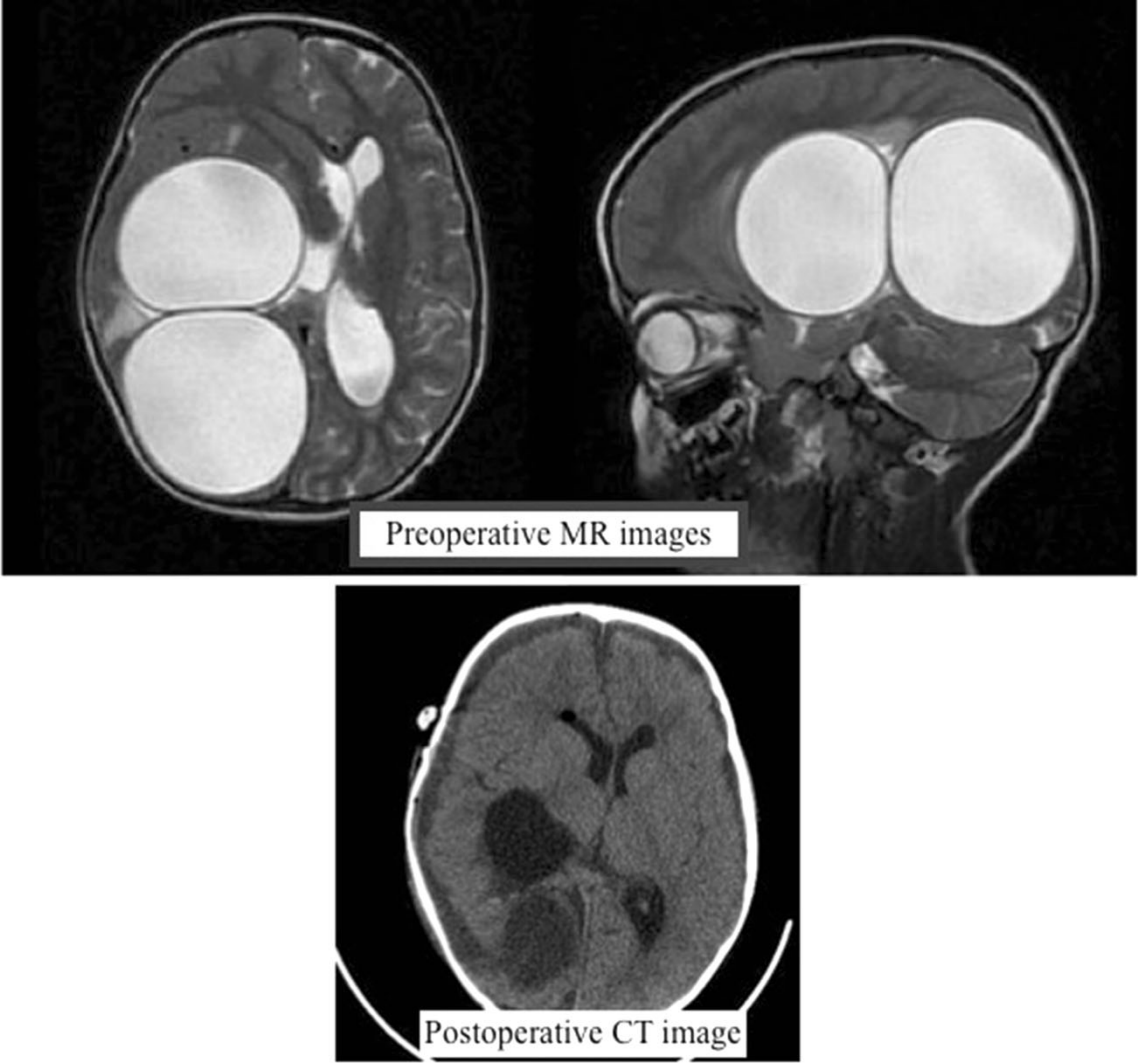
T2-weighted MR image of a smooth-edged, 6-cm diameter hydatid cyst in the right temporal-parietal lobe, with a second smooth-edged 7-cm diameter hydatid cyst starting in the right parietal lobe nearly filling the occipital lobe, anterior and neighbouring the first, exceeding the occipital cortex to the posterior. Cranial CT check after removal of both hydatid cysts, showing reduction in the cyst location and mass effect of the cysts

**Fig. 3:**
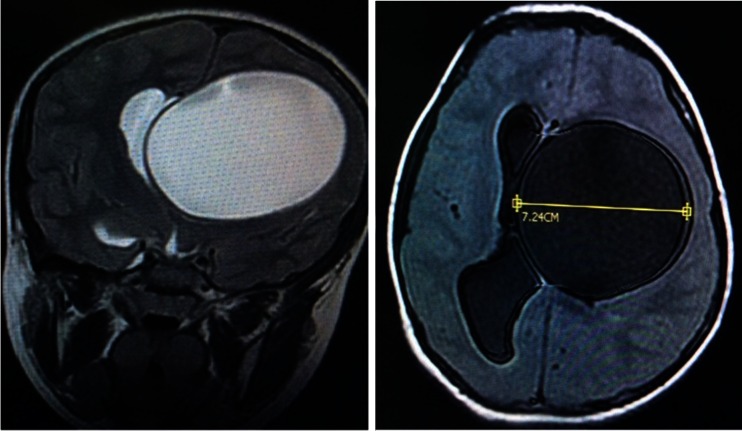
Axial and coronal images of a large hydatid cyst causing a shift located in the deep left temporoparietal was revealed

This study evaluates the balloon-assisted dissection technique we developed in the removal of four large hydatid cysts in three patients (Figs. 1, 2 and 3). We believe that the balloon-assisted technique is a new method that increases the chances of removing hydatid cysts without rupture.

## Relevant surgical anatomy

As the hydatid cyst can form in any anatomical region within the cranium or brain, a standard surgical anatomy description was not completed.

On preoperative radiological tests, more complicated adhesions that may ease cyst rupture, such as local calcification of the cyst wall or vascularisation in the cleavage plane, may be suspected. It is recommended that the Foley probe be inflated a little away from these areas, accompanied by intraoperative ultrasound. In this way it may be possible for the cyst wall to be passively removed from the adhesion bands.

## Description of the technique

**Fig. 4:**
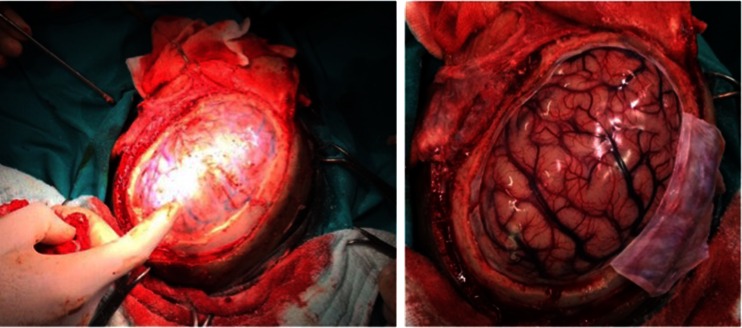
Wide craniotomy and dura opening

The new surgical method we developed is applied during classic wide craniotomy for hydatid cyst surgery or standard surgical interventions like wide dura opening (Fig. 4). It was determined that the region where the cyst comes out of the cortex, or where the cortex is thinnest, is the most appropriate place to deliver the cyst. Firstly, dissection between the cyst and brain tissue was done to allow the tip and uninflated section of the balloon of the Foley probe to enter without penetrating the parenchyma. Starting from the surface, the Foley probe was advanced step by step around the cyst towards the base, and in regions where dissection was required, the balloon of the Foley probe was inflated and dissection was completed to free any adhesion bands.

Firstly a number 12 Foley probe was advanced between the cyst and brain, the balloon was inflated with saline and the hydatid cyst was freed from the brain tissue. When the cleavage line was formed, to ease delivery the Foley probe was increased to numbers 14 and 16. To start delivery of the cyst, the balloon was inflated with water in stages and the balloon was pulled slightly outward and released to support the initiation of the cyst removal. During each inflation, we patiently waited for the balloon to push the cyst slightly outward, allowing gravity and, if necessary, a finger on the surface of the cyst to slowly roll the cyst outward and ease removal. This method was used without requiring irrigation with hypertonic saline to remove four large hydatid cysts in three cases without damaging the cyst wall, allowing dissection and delivery without rupture (*see* ESM 1 movie).

In the early postoperative period, the patient is monitored in the intensive care unit with the Glasgow Coma Scale for neurological follow-up. The surgical field is checked with postoperative cranial computerised tomography (CT)

## Indications

We believe the balloon-assisted hydatid cyst removal method may be used to remove large cerebral hydatid cysts exceeding the cortex or located more deeply without rupture.

## Limitations

We believe this method is not appropriate for hydatid cysts with calcified walls or infection. If the most distal tip of the balloon of the Foley probe penetrates brain parenchyma and the balloon is inflated, brain damage may occur. Dowling’s method has similar risks. In fact, if the irrigation probe penetrates the parenchyma and is not noticed, uncontrolled administration of fluid may cause more harmful unwanted complications. Technically, if the balloon of the Foley probe is used, a balloon apparatus that is easier to use and more effective, which also allows irrigation, may be developed. To determine the true success rate of the technique, there is a need to increase the number of cases.

## How to avoid complications

To deliver hydatid cysts successfully and easily, preoperative radiological examination should be completed to evaluate alveolar type, *Echinococcus* infection and cyst wall calcification. The number, location, distance to surface and most appropriate intervention location of the hydatid cysts should be determined. Wide craniotomy and dura opening allow both easy removal of the cyst and the possibility to access different intervention locations. The most significant complication of cerebral hydatid cyst surgery is rupture of the cyst. In case of a situation with cyst rupture, hypertonic saline should be prepared and ready to wash the interior of the cyst and surrounding tissue. We believe that patient application of this method may prevent development of this complication.

Additionally, the slow inflation of the balloon in stages both eases dissection and exposes the cerebral parenchyma to less pressure.

## Specific peri-operative considerations

The most important factors are careful dissection, slow inflation of the balloon in stages and patient waiting for the hydatid cyst to deliver, changing table position to gain more benefit from gravity and the valsalva manoeuvre to remove the cyst without rupture.

To see that the Foley probe is advancing correctly, to identify the areas of intense adhesion, to determine anatomical relations and to monitor the inflating balloon, the use of intraoperative ultrasound can provide significant contribution to preventing possible complications.

## Specific information to give to the patient about surgery and potential risks

This method has been improved from Dowling’s method. We use a balloon instead of saline irrigation. Patients can be informed that potential risks do not differ from the standard Dowling method.

## The key points

1. Hydatid cyst cases with no complications should be chosen
2. Surgical planning should be completed together with preoperative careful radiological and clinical evaluation
3. Wide craniotomy and dura opening should be performed
4. Primarily the cyst should be reached by the shortest route with least neural damage and the initial dissection should not weaken the cyst wall
5. After initial dissection, taking care that the distal tip of the Foley probe does not penetrate the brain parenchyma, the balloon should be slowly inflated. Advancing step by step, the dissection and delivery process should begin.
6. The area where the cyst comes out of the cortex should be used as the delivery window and the delivery process should be patiently allowed to proceed slowly; if necessary, the process can be supported by gently rolling it out with a finger
7. Though not necessary in our cases, if required it is possible to benefit from the dissection effect of physiological serum and water
8. Due to the risk of rupture of the cyst wall, hypertonic saline should be prepared and the area should be washed with hypertonic saline
9. To benefit more from gravity, if necessary the table position should be changed
10. The dura should be tightly closed and postoperative radiological checks and infectious disease monitoring should be provided

## Electronic supplementary material

ESM 1 (M4V 73779 kb)
